# Balancing nature and technology: enhancing flowering in ornamental plants

**DOI:** 10.1186/s40529-025-00487-7

**Published:** 2025-12-16

**Authors:** Zhen-Rong Cai, Dewi Sukma, Ming-Tsair Chan

**Affiliations:** 1 Academia Sinica Biotechnology Center in Southern Taiwan, Agricultural Biotechnology Research Center, Academia Sinica, Tainan, 71110 Taiwan; 2 Department of Agronomy & Horticulture, Faculty of Agriculture, IPB University, Bogor, 16680 Indonesia; 3 Graduate Program of Translational Agricultural Sciences, National Cheng Kung University and Academia Sinica, Tainan, 70101 Taiwan

**Keywords:** Environmental factors, Flowering regulation, Gene editing, Genetic engineering, Ornamental plants, Smart agriculture

## Abstract

Commercial ornamental plant growers require consistent flowering to adequately fulfill the needs of the industry in the global markets. Combining traditional plant breeding practices with contemporary technology-based solutions, such as eco-friendly methods and AI-focused models, is the way forward for plant flowering and production optimization to attain quality and sustainability. This review discusses the various factors that can be harnessed to modulate plant flowering. These include the major genetic flowering control mechanisms in ornamental plants, such as the key flowering time genes like *Flowering Locus T* (*FT*) and *CO* (*CONSTANS*) that make up genetic circuits responding to various factors such as hormones (e.g. ABA, gibberellins), as well as key environmental signals like light/dark and temperature. In tandem, the application of plant hormones, like auxins and gibberellins, provides a hormonal approach to enhance flower formation and yield physiologically. Furthermore, innovative improvements such as genetic editing through tools like overexpression or CRISPR/Cas9 and smart systems with sensors that measure parameters and automate controls are now crucial to improving outcomes. However, challenges still exist, including genetic variability and resource limitations, highlighting the necessity for adaptive approaches to sustainable horticulture. Combining these traditional and modern approaches will improve flowering traits and realize the cultural value of ornamental crops in the changing world economy.

## Introduction

Ornamental plants are economically important in producer nations and play roles in urban planning and horticulture. For example, in Taiwan in 2022, the production value of ornamental plants and flowers was NTD8.5 billion (https://www.tcdares.gov.tw/en/ws.php?id=8881), highlighting the industry’s growing importance. Overall, the world flower and ornamental plant market was valued at USD43.09 billion in 2023 and is expected to reach USD67.56 billion by the end of 2028, registering a compound annual growth rate (CAGR) of 9.7% (Smith 2024).

Ornamental plants are integral to landscape design, where they beautify outdoor spaces such as gardens, parks, and public spaces and also provide better air quality and peaceful environments that encourage community health and positively impact the quality of life (Marcus and Francis 1997; Zhuang et al. 2021; Gush et al. 2024). Furthermore, the inclusion of these plants has important ecological consequences, supporting biodiversity by providing resources to pollinators for ecological system stability (Katumo et al. 2022).

For ornamental plant cultivation to be commercially viable, it needs to produce high-quality flowers that are attractive to consumers and support markets that will push economic development (Haviland-Jones et al. 2005). Repeat and exuberant flowering of oriental plants is a mandatory requirement for market success with the public.

Natural methods used to modulate flowering constitute manipulation of the plant interface and the environment (Al-Shammary et al. 2024). In recent years, genetic engineering, light technology, and controllable climate have changed the scale of flower breeding to enhance yields, quality and reliance on natural cycles (Miller and Silva 2018; Karanisa et al. 2022). The integration of traditional techniques with modern technologies balances economic benefits with ecological preservation while achieving aesthetic objectives (Wani et al. 2023). Production countries have invested consistently in these field to stimulate rapid and optimal growth, flowering, and postharvest freshness (Noman et al. 2017).

In this review, biological and environmental factors, as well as technical advances that influence flowering in ornamental plants, are presented. These factors are examined individually to better understand their roles in optimizing flower production. Additionally, insights into potential applications are discussed.

## Biological factors

Genetics is a fundamental determinant of flowering behavior in ornamental plants, governing their transition from vegetative to reproductive growth, a critical process for initiating flowering. Key genes, such as *Flowering Locus T* (*FT*) and *CONSTANS* (*CO*), have been identified as central regulators of this process (Wang et al. 2024). These genes interact with environmental factors like day length and temperature, ensuring that flowering occurs under optimal conditions. In contrast, late-flowering cultivars may possess genetic traits that delay this transition, allowing them to bloom later in the season (Alonso Segura and Socias 2017). This genetic diversity is invaluable for horticultural practices, facilitating the selection of cultivars tailored to specific environmental conditions or market needs. The literature underscores the importance of these genetic mechanisms in synchronizing flowering with external cues (Andrés and Coupland 2012). For example, the diversity and selection of the continuous-flowering gene *RoKSN* in roses plays a significant role in breeding programs designed to extend flowering periods (Soufflet-Freslon et al. 2021). Understanding and utilizing these genetic differences will improve both the aesthetic and economic value of ornamental plants.

The plant hormones (auxin and cytokinin) work synergistically to ensure proper flower formation and maturation (Matías-Hernández et al. 2015; Cucinotta et al. 2021). For instance, auxins promote cell elongation and differentiation, whereas cytokinins are involved in cell division and differentiation, balancing the growth processes necessary for flower formation (Chandel et al. 2023). Some of these plant growth regulators have been shown to induce floral production in ornamental plants (Lee et al. 2021). The cytokinin analog BAP promotes cell division and differentiation,to which enhances flower formation by mimicking the effects of natural cytokinins, increasing flower yields and improving the quality of blooms (Blanchard and Runkle 2008). Furthermore, exogenous gibberellic acid (GA) application accelerates the transition from vegetative to reproductive growth, leading to earlier flower production (Lee et al. 2021). Exogenous application of GA can break dormancy and induce early flowering in plants such as *Chrysanthemum morifolium*, meeting market demands for specific flowering periods (Dong et al. 2017). In *Rosa* spp., GA treatments increased flower size and number GA facilitates the transitio from vegetative to reproductive phases by activating flowering-promoting genes such as *Leafy* (*LFY*) (Blazquez et al. 1998). Additionally, GA interacts with the flowering-time gene *Suppressor of overexpression of constans 1* (*SOC1*), integrating environmental signals to control flowering onset (Moon et al. 2003).

## Environmental factors

Environmental factors play a significant role in influencing the flowering of ornamental plants. Understanding these external influences is crucial for horticultural practices and optimizing plant growth. For instance, photoperiodism refers to the plant’s response to the duration of light, which determines plant flowering time (Ha 2014). The light-dependent mechanism (long days or short days) determines the timing of flower production across different species (Smith 2024). Short-day plants, such as chrysanthemums (*Chrysanthemum morifolium*), require extended periods of darkness to initiate flowering and naturally bloom in the fall, allowing for year-round production that meets market demands (Higuchi 2018). Long-day plants like lettuce (*Lactuca sativa*) need shorter nights; therefore, providing supplemental lighting to extend day length induces flowering, enhancing off-season production (Lee et al. 2024).

Temperature is a critical factor influencing the flowering of horticultural crops (Cockshull et al. 1981). Vernalization, a period of prolonged cold temperatures, is for certain plants to initiate flowering (Amasino 2004). Horticulturists apply artificial vernalization techniques, such as in tulip bulbs, which is particularly useful for meeting market demands and extending the growing season (Ha 2014). To promote flowering initiation, mature *Phalaenopsis* orchids should be cultivated in ambient temperatures of 24 °C during the day and 18 °C at night (Blanchard and Runkle 2006). Conversely, some plants may experience delayed flowering or floral abnormalities when exposed to unfavorable temperatures, highlighting the importance of temperature management in cultivation practices (Zinn et al. 2010). By controlling greenhouse temperatures, growers can optimize flowering, improve flower quality, and increase yield (Wani et al. 2023).

**Fig. 1:**
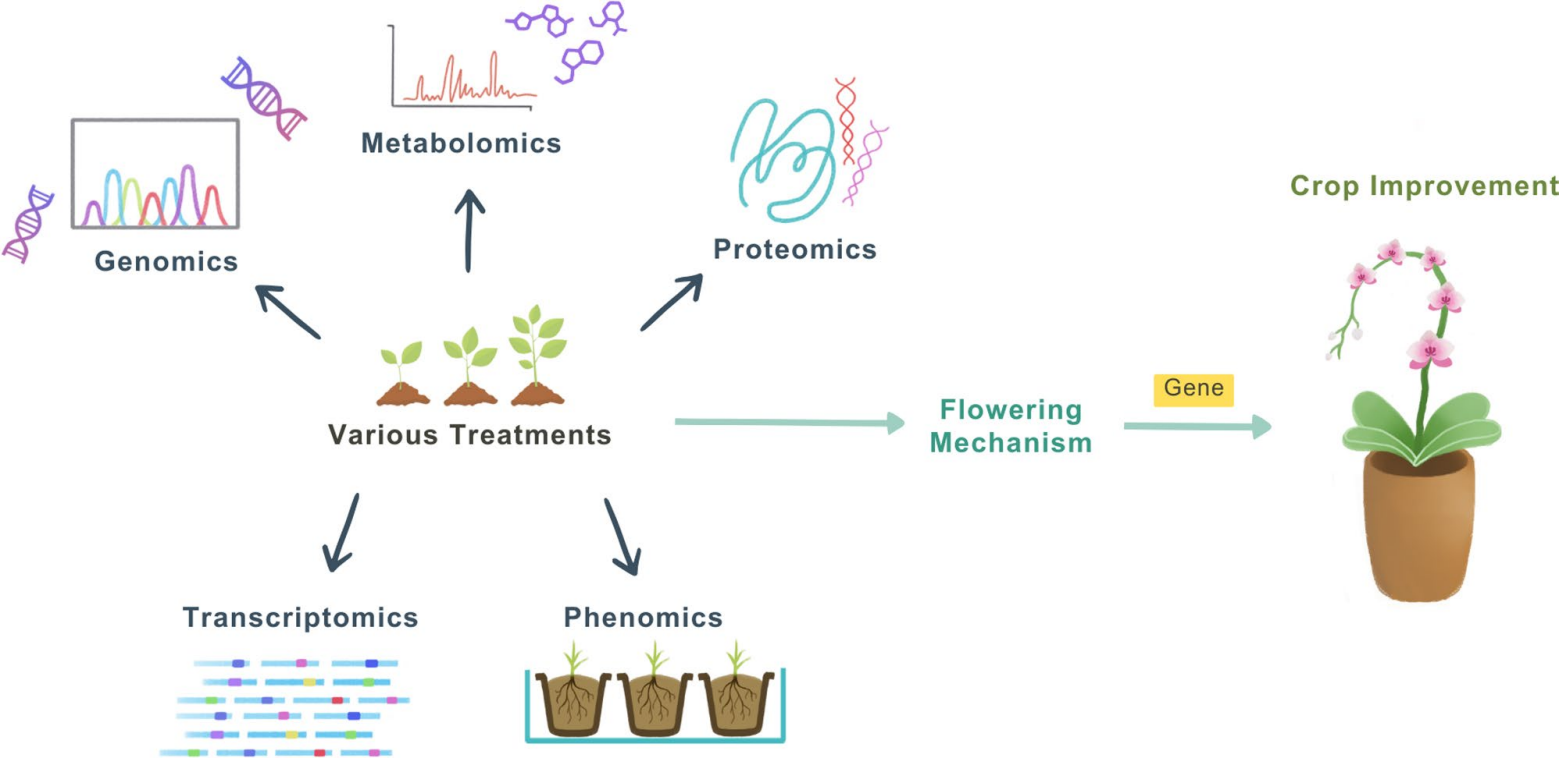
Using multi-omics for deepening understanding of flowering in ornamental plants. Multi-omics approaches are important for dissecting the molecular mechanisms controlling flowering and mining for potential candidate genes that control this central process of life

Water and nutrient availability are critical factors influencing the flowering of horticultural crops. Adequate water supply ensures proper cell turgor, nutrient transport, and metabolic activities that support flower development (Beauzamy et al. 2014). For example, drought stress can promote flowering in ornamental crops like petunias (*Petunia* × *hybrida*) but reduce flower size, negatively impacting the aesthetic quality (Tran et al. 2024). In certain species, controlled water-deficit strategies, such as those used with poinsettias (*Euphorbia pulcherrima*), are employed to promote earlier flowering and improve ornamental traits (Caser et al. 2017). Nutrient availability, particularly nitrogen (N), phosphorus (P), and potassium (K), plays a significant role in the regulation of flowering (Zhang et al. 2023). Adequate nitrogen and phosphorus are important for flower initiation; phosphorus deficiency can delay flowering and reduce flower number in species like marigolds (*Tagetes erecta*) (Naik 2015). In roses (*Rosa* spp.), balanced fertilization with appropriate levels of potassium enhances flower color intensity and prolongs vase life (Yousefi et al. 2019). To gain a deeper understanding of the factors influencing flowering, researchers can employ approaches such as high-throughput phenotypic analysis, genome and transcriptome comparisons, as well as phenomics, proteomics and metabolomics to conduct in-depth analyses. These methods enable comparisons of gene expression to explore the mechanisms behind flowering (Fig. 1).

## Technological advances in ornamental plant flowering

Modern technologies are implemented in horticulture to automate the flowering of ornamentals, thereby deviating from traditional approaches. Innovations also contribute to flower quality and quantity, and ensure that environmental factors that impact plant development impose less control over plants. To date, research into ornamental plants has made use of multiomics that give more insight into plant genetics, physiology, and ecology, understanding of which is imperative for both basic science and commercial applications in horticulture (Fig. 1). Whole-genome sequencing and other genomic approaches allow identification of polymorphisms, markers linked to desired traits, to describe the comprehensive genetic landscape of ornamental plants such as gene expression patterns and transcriptomics, at different developmental stages or when the plant is under stress. Proteomics is working towards identifying proteins and their functional roles in ornamental plants by profiling (Kosová et al. 2021). Metabolomics focuses on metabolite profiling to study small molecules within plants and elucidate metabolic pathways associated with color, scent, and stress signals. In general, multi-omics uses integrative genomics, transcriptomics, proteomics and metabolomics tools to assess the flowering effects. As in all plants, biological processes are combined in ornamental plants. Omics approaches facilitate the elucidation of connections between specific genes and their phenotypic traits as well as their responses to the environment (Luo et al. 2024).

More studies using multi-omics approaches will be beneficial for improving plant qualities such as floral longevity, disease resistance and climate stress tolerance in the vegetation form. Phenotyping employs high-throughput phenotyping using automated equipment and imaging technologies to measure a wide array of plant traits such as growth, number of flowers, and stress responses from high-throughput studies (Cheng et al. 2025). Direct field studies in natural habitats can determine the ecological interactions and environmental factors that affect ornamental plant growth and health. Integration of these cutting-edge approaches, primarily through multiomics, is revolutionizing ornamental plant research to facilitate breeding, conservation, and sustainable horticulture (Sarfraz et al. 2025). Furthermore, these tools allow for local target validation of candidate genes by virus-induced gene silencing (VIGS), a transient knockdown method, to test function. Biochemically the candidate gene(s) can be attained by loss-of-function, chimeric repressor gene silencing technology (CRES-T) (Mitsuda et al. 2011), RNA interference (RNAi) (Hung and Slotkin 2021), clustered regularly interspaced short palindromic repeats (CRISPR) (Tuncel et al. 2025), or gain-of-function, or overexpression of the specified genes to manipulate flowering characteristics. We can improve flowering using these state-of-the-art genetic manipulation tools, allowing ornamental plant variation with the desired traits.

Recent advances in biotechnology mean that ornamental plant flowering traits can now be improved by directly manipulating their genetic material (Chandler and Sanchez 2012). Scientists can make changes to the genes that control flower size, color, scent, and flowering time by genome engineering. For instance, researchers have created blue carnations and roses by inserting the genes encoding for delphinidin pigment biosynthesis into these species (Katsumoto et al. 2007). Overexpression of a mutant ethylene receptor gene mDG-ERS1 achieved early flowering in chrysanthemums (Morita et al. 2012). Overexpression of the gene *CmFTL3* has been shown to be an early flowering inducer in chrysanthemums (Oda et al. 2011). These notable genetic advancements shorten the juvenile phase, allowing breeders to save time breeding new cultivars. The commercial value of these innovations, driven by consumer desire to purchase something different and novel, can be significant. Overexpression of the *NFL1* gene, similar to the tobacco LEAFY-like gene, promotes early flowering in transgenic petunia (Ahearn et al. 2001). Early flowering in the cultivar *Dendrobium* Chao Praya Smile has been induced by overexpression of the *DOAP1* gene (Sawettalake et al. 2017). The impact of this genetic modification is particularly favorable in orchids, which generally take longer to shed their juvenile phase, as it quickens the breeding and commercialization of new strains. This facilitates faster selection actions and leads to the creation of new ornamental lines with traits of high interest.

CRES-T utilizes a chimeric repressor by combining a transcription factor with the plant-specific EAR-motif repression domain (SRDX). This system predominantly suppresses target genes regulated by the transcription factor, effectively overriding the activity of endogenous and functionally redundant transcription factors (Hiratsu et al. 2003). The resulting plant phenotypes from the CRES-T system resemble those of mutants with a loss-of-function mutation in the corresponding transcription factor gene. CRES-T serves as a straightforward and powerful tool for functional gene analysis and for manipulating plant traits through precise gene expression repression (Mitsuda et al. 2011). A chimeric repressor of GtMYB3 modified blue-flowered gentian into two transgenic lines with picotee phenotypes, reduced anthocyanin, increased flavone levels, suppressed late flavonoid biosynthetic genes, and increased the potential for creating novel flower patterns (Nakatsuka et al. 2011). Creating a “loss-of-function” phenotype is beneficial for studying the role of transcription factors and can also be applied to modify plant traits, including flower color and shape (Sasaki 2018).

RNAi serves as a molecular mechanism for suppressing target genes. This process begins with the degradation of double-stranded RNA (dsRNA) into small interfering RNAs (siRNAs) through an RNase III-like activity, which subsequently forms the RNA-induced silencing complex (RISC) to degrade mRNA (Agrawal et al. 2003). RNAi has been demonstrated to effectively promote flowering time by inactivating floral repressors. For example, the long noncoding RNA lncWD83 in roses enhances the ubiquitination of the floral repressor RxMYC2L and thereby accelerates flowering time (Yeqing et al. 2023). The orchestration of flowering regulation by noncoding RNAs highlights their potential applications in plant improvement and breeding strategies (Liu et al. 2023). A novel approach in plant biotechnology, known as spray-induced gene silencing (SIGS), involves the application of RNA solutions—such as double-stranded RNA (dsRNA), hairpin RNA (hpRNA), small interfering RNA (siRNA), or microRNA (miRNA)—directly onto plant surfaces to trigger RNAi-mediated silencing of specific genes within the plant (Dubrovina et al. 2025). Further research is necessary to refine methods for silencing floral repressors in order to expedite flowering initiation in plants.

**Fig. 2:**
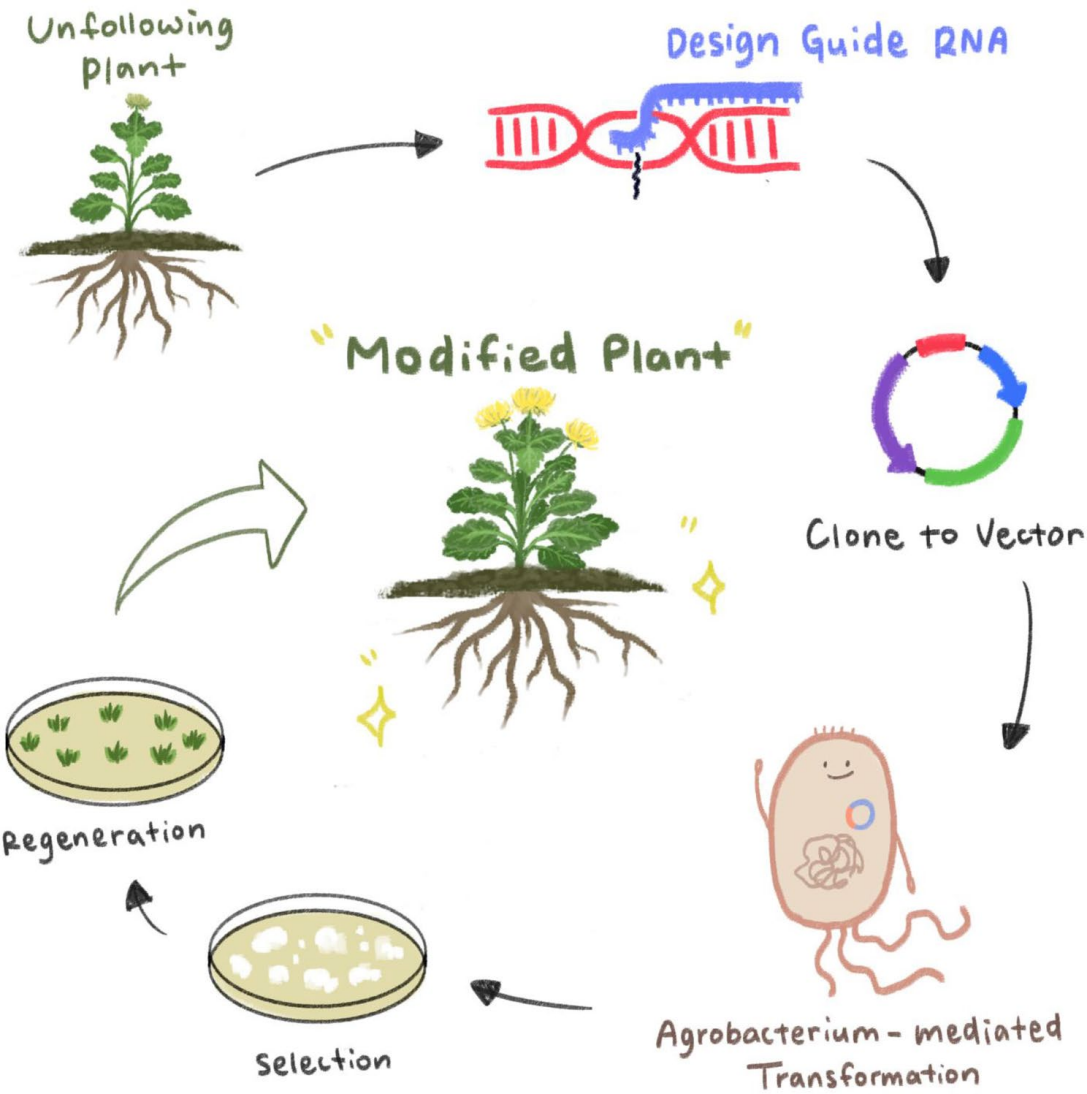
Using CRISPR/Cas9 as a gene editing strategy in ornamental plants. Wild-type plants are used as targets, and guide RNAs are designed according to the gene sequence to be modified. These are cloned into the vector, and agrobacterium is used for transformation. After screening and regeneration steps, plants with controlled flowering are obtained

Advanced biotechnological techniques, including gene editing with CRISPR/Cas9, have transformed ornamental plant breeding (Saini et al. 2023). These methods enable precise alterations of specific genes to improve traits such as disease resistance, stress tolerance, and flowering time. By targeting genes associated with the flowering process, CRISPR/Cas9 makes it possible to adjust flowering time and modify flower structures (Fig. 2). For instance, knocking out genes that inhibit flowering can lead to earlier blooming, whereas editing genes that promote flowering can delay it or modify floral characteristics (Zhu et al. 2020). CRISPR/Cas9 is a cutting-edge genome-editing tool that allows for highly accurate modifications, facilitating the regulation of traits like flowering time and floral morphology (Liu et al. 2024). In addition, it is possible to knock out a gene without leaving any foreign gene sequence. The transgene-free concept is supported because self-crossing eliminates the selection marker gene in subsequent generations. These advanced CRISPR plants have the potential to quell concerns surrounding genetically modified organisms (GMOs).

In ornamental plants, manipulating flowering traits can greatly enhance their aesthetic appeal and commercial value. While much of the research has focused on model plants and crops, the application of CRISPR/Cas9 in ornamental plants is steadily growing. For example, editing the *TERMINAL FLOWER* (*TFL*) gene, a known flowering repressor, has been demonstrated to influence flowering time in various species. Silencing the *PhTFL1* gene produced early flowering phenotypes in petunia plants (Abdulla et al. 2024). Similarly, the ectopic expression of *RoKSN*, a *TFL1* homolog, suppressed flowering in roses (*Rosa hybrida*) (Randoux et al. 2014). This genetic modification extends the juvenile phase in roses, supporting accelerated breeding programs and the creation of novel ornamental cultivars. These findings highlight that knocking out repressor genes using CRISPR/Cas9 can enhance flowering in ornamental plants.

Recent studies have already found many repressor genes in various ornamental species (Table 1). These repressor genes might also be potential targets, allowing CRISPR/Cas9 to create early-flowering ornamental plants. These results also imply that knocking out the repressor(s) may have led to early flowering phenomena enabling escape from the regulation of environmental or biological factors. This technology can shorten the juvenile stage of long-developing ornamental plants such as *Fagus sylvatica* and *Malus sylvestris,* thereby promoting their breeding. It might even help conserve endangered plant species such as *Rhododendron kanehirae* (Schippmann 2020). Overall, CRISPR/Cas9 provides a precise and effective tool for regulating flowering traits in ornamental plants. By leveraging targeted gene editing, breeders can develop new varieties with specific flowering characteristics, catering to market demands and increasing the ornamental value of these plants. Conventional CRISPR techniques are not suitable for studying essential genes, as deleting these genes is lethal to the cell or organism. However, this challenge can be addressed using a modified CRISPR approach called CRISPR inhibition (CRISPRi). CRISPRi employs a catalytically inactive Cas9 protein (dead Cas9) paired with a guide RNA (gRNA) to regulate transcriptional activity at the DNA level (Ghosh et al., 2016). In addition, RNAi can be another alternative method to knock down the target gene. The choice between CRISPR and RNAi largely depends on the experimental goal. For some studies, partial gene knockdown may be more appropriate to answer specific research questions, while others may require a complete knockout to eliminate even trace amounts of functional mRNA that could sustain biological activity.

**Table 1:** Flowering repressors in ornamentals (References 2014–2025)

Plants	Repressor(s)	References
Chrysanthemum	*CmFTL2* is a flowering repressor gene	(Sun et al. 2017)
Chrysanthemum	*CsAFT*, an anti-florigenic FT/TFL1 family protein, is a repressor	(Oda et al. 2020)
Chrysanthemum	*CmRCD1* is a flowering repressor gene	(Wang et al. 2021)
Chrysanthemum	CmSVP and TPL are corepressors inhibiting chrysanthemum flowering	(Zhang et al. 2023)
Chrysanthemum	CmNRRa and Cm14-3–3μ work synergistically as corepressors in chrysanthemum flowering	(Cheng et al. 2023)
Chrysanthemum	*CmTEM1*, TEMPRANILLO1, inhibits flowering	(Cheng et al. 2023)
Chrysanthemum	*CmERF3*, an ethylene-responsive transcription factor, controls vegetative growth	(Cheng et al. 2023)
Chrysanthemum	*CmERF3*, an ethylene-responsive transcription factor, controls vegetative growth	(Cheng et al. 2023)
Chrysanthemum	*CmBBX5*, a B-box subgroup I member comprising two B-boxes and a CCT domain, controls vegetative growth	(Wang et al. 2024)
Chrysanthemum	*CmFLC-like*, a MADS-box transcription factor, represses flowering under low temperature	(Hu et al. 2025)
Iris	*IgSVP*, *SHORT VEGETATIVE PHASE,* and *IgTFL1*, *TERMINAL FLOWER 1* may be repressors	(Fan et al. 2023)
Orchid (Dendrobium)	*DnSIZ1*, a SIZ/PIAS-type SUMO E3 ligase gene, may be a negative regulator of flowering	(Liu et al. 2015)
Orchid (*Dendrobium catenatum*)	*DeHd3b* may function as a repressor	(Zheng et al. 2019)
Neotropical orchids	*MonFT1A* and *MonF1B*, similar to *TFL1-like* gene, function as repressors	(Ospina-Zapata et al. 2020)
Orchid (Dendrobium)	*DOTFL1*, *TERMINAL FLOWER1,* promotes vegetative growth and suppresses orchid flowering	(Li et al. 2021)
Petunia	*PhTFL1* is a repressor	(Wu et al. 2019)
Orchid (Phalaenopsis)	*FLOWERING LOCUS T*-like gene showed a role as a repressor on spike initiation	(Lu et al. 2024)
Rose	*RoKSN* gene is a negative control for flowering	(Randoux et al. 2014)
Rose	RcPIFs, phytochrome-interacting factors, interact with RcCO to inhibit RcFT expression under low light	(Sun et al. 2021)
Rose	*RcMYC2L* is a repressor of flowering	(Yeqing et al. 2023)
Rose	*RhLHY*, LATE ELONGATED HYPOCOTYL, and *RhTPPF*, Trehalose-6-phosphate phosphatase F, are repressors under low light conditions	(Fan et al. 2025)

Smart systems in horticulture can precisely modulate environmental parameters such as lighting, temperature, humidity, and nutrient uptake (Nelson and Bugbee 2014). By combining sensors and automation, it is possible to establish an optimal environment for flower initiation that, in turn, increases yield and quality. LED grow light systems have been developed to deliver precise light spectra that can be managed in LED arrays to induce flowering of greenhouse-grown ornamentals (Bourget 2008). Automated climate control systems have been used for the synchronous flowering in orchids (*Orchidaceae*) (Lopez and Runkle 2005). Hydroponics, including feedback on nutrients, allows for precise nutrient supply, optimizing plant health (Fathidarehnijeh et al. 2023). These technological advancements contribute to the efficient production of ornamental plants with desirable flowering characteristics. By integrating genetic engineering, biotechnology techniques, and smart growing technologies, breeders can meet consumer demands and enhance the economic value of ornamental plants.

## Challenges and limitations

Despite significant advancements, some hurdles remain in order to optimize flowering in ornamental crops. Changing environmental conditions cause substantial threats to the synchrony of flower production (Borghi and Perez 2019). Different environmental factors, such as varying temperatures and unpredictable weather conditions, can potentially disrupt the natural growth of plants, leading to flower outputs that differ crop-by-crop. Furthermore, plant species have inherent genetic variability and, therefore, do not show uniform traits in breeding. The wide genetic diversity impedes the transference and stabilization of desired traits among clones, which is a challenge for consistent and dependable flower production (Govindaraj et al. 2015). Resource allocation also presents a significant barrier. Limited funding and human resources for research and technology implementation restrict the scope and pace of advancements in this field (Sartas et al. 2020). Insufficient financial and personnel support slows the development, making it more challenging to address existing issues (Suparno et al. 2023).

To address these challenges, adaptive strategies are crucial. These include developing resilient plant varieties, fostering collaborative research efforts, and increasing investment in research initiatives. Such approaches can help manage plant flowering sustainably under evolving environmental conditions. For instance, environmental modeling has emerged as a valuable tool. Researchers have developed computer models capable of predicting and optimizing flower production based on environmental factors. These predictive models assist horticulturists in determining the ideal environmental conditions for specific plant species, thereby, enhancing flower yield and quality (Lan et al. 2022).

## Future directions

Emerging trends and potential research areas present promising opportunities to tackle current challenges in ornamental horticulture. Sustainable practices, such as using eco-friendly methods to boost flower production without harming the environment, are drawing increasing attention (Coulibaly et al. 2021). Key strategies include adopting organic farming techniques, enhancing water efficiency, and minimizing the use of chemical fertilizers and pesticides. Additionally, integrating beneficial microbes, insects, and natural repellents can significantly reduce dependence on synthetic pesticides while maintaining high flower quality.

Cross-disciplinary collaboration is critical for driving innovation in horticulture. Combining expertise from genetics, environmental science, and engineering can yield transformative solutions. For instance, integrating genetic research with environmental monitoring can help develop resilient plant varieties that thrive under changing climatic conditions. Moreover, the adoption of advanced digital technologies, such as artificial intelligence (AI) and the Internet of Things (IoT), is poised to revolutionize optimizing and monitoring environmental factors to improve flower outcomes. AI-driven models, for example, have the potential to predict optimal planting times and adjust environmental variables in real-time, maximizing both flower production and quality (Al-Qudah et al. 2025).

Plant biotechnology demands simple and accurate gene editing that does not involve foreign DNA transfer to the genome. The literature already reports methods, including base editing and prime editing, to achieve site-specific genomic editing with no transgene integration (Gu et al. 2021). This is particularly useful in ornamental plants, because consumer acceptance is largely dependent on the use of transgenic DNA. Recently, it was found that another CRISPR/Cas9 method without transgenes can be integrated into plants, which delivers mobile CRISPR/Cas9 RNA from roots to shoots and flowers without any transgenes (Yang et al. 2023). Another strategy is to create a miniature CRISPR vector from tobacco rattle virus (TRV) to deliver the compact RNA-guided TnpB enzyme ISYmu1, and its guide RNA (Weiss et al. 2025).

These innovative approaches provide a path around the regulatory impediments of transgenic plants and hold promise for faster breeding of commercial varieties with desirable traits while bolstering public trust in biotechnology (Garland and Curry 2022). By prioritizing these directions, researchers can overcome current limitations and enhance the performance of ornamental plants, paving the way for remarkable progress in the field of horticulture.

## Conclusion

Integrating traditional practices and technological advancements offers numerous benefits for sustainable and efficient flower production in ornamental plants. Combining natural resilience with technological precision can lead to flexible and productive plant systems. However, challenges remain, particularly in ensuring long-term environmental sustainability. Balancing technological enhancements with ecological considerations is essential to prevent adverse effects on ecosystems (Gamage et al. 2024).

Understanding and managing the factors that influence flowering in ornamental plants is necessary for both aesthetic appeal and economic viability. Biological and environmental aspects, including genetic studies and environmental modeling, play a critical role in determining the visual and commercial success of these plants. Consistently producing attractive flowers is important for enhancing their beauty and for driving economic growth in the horticulture sector. Recent research provides valuable insights into addressing challenges in plant flowering, especially regarding the significance of genetic factors in flower development (Cai et al. 2024), and the impact of environmental conditions on flower production (Cho et al. 2017). Artificial Intelligence (AI) and the Internet of Things (IoT) are transforming agriculture by facilitating precise monitoring and optimization of environmental factors, thereby improving flowering outcomes. AI algorithms analyze data from IoT sensors to enable real-time adjustments to parameters like temperature, humidity, and light intensity, ensuring optimal conditions for plant growth (Shamshiri et al. 2018). Machine learning models further enhance this process by analyzing historical data and environmental patterns to predict the best flowering times, allowing growers to take timely actions for improved results (Liu et al. 2022). Additionally, IoT devices monitor soil moisture and nutrient levels, providing critical data for AI systems to optimize irrigation and fertilization practices, which are essential for healthy flowering (Goap et al. 2018). The integration of AI and IoT not only enhances precision in environmental control but also results in healthier plants and higher flower yields, promoting better resource management and agricultural productivity (Bishnoi et al. 2024). Fostering interdisciplinary efforts and leveraging modern tools will be crucial for overcoming current limitations and driving innovation in ornamental plant flowering.

## Data Availability

No supplementary data are available.
